# Ultrasound-mediated microbubbles cavitation enhanced chemotherapy of advanced prostate cancer by increasing the permeability of blood-prostate barrier

**DOI:** 10.1016/j.tranon.2021.101177

**Published:** 2021-07-13

**Authors:** Haizhui Xia, Decao Yang, Wei He, Xuehua Zhu, Ye Yan, Zenan Liu, Tong Liu, Jianling Yang, Shi Tan, Jie Jiang, Xiaofei Hou, Huile Gao, Ling Ni, Jian Lu

**Affiliations:** a Department of Urology, Peking University Third Hospital, Beijing 100191, China; b Institute of Medical Innovation and Research, Peking University Third Hospital, Beijing 100191, China; c Department of Ultrasound, Peking University Third Hospital, Beijing 100191, China; d Key Laboratory of Drug-Targeting and Drug Delivery System of the Education Ministry, Sichuan Engineering Laboratory for Plant-Sourced Drug and Sichuan Research Center for Drug Precision Industrial Technology, West China School of Pharmacy, Sichuan University, Chengdu 610064, China; e Institute for Immunology and School of Medicine, Tsinghua University, Medical Research Building, Beijing 100084, China

**Keywords:** Prostate cancer, Chemotherapy, Ultrasound, Cavitation, Immune

## Abstract

Although chemotherapy is an important treatment for advanced prostate cancer, its efficacy is relatively limited. Ultrasound-induced cavitation plays an important role in drug delivery and gene transfection. However, whether cavitation can improve the efficacy of chemotherapy for prostate cancer remains unclear. In this study, we treated RM-1 mouse prostate carcinoma cells with a combination of ultrasound-mediated microbubble cavitation and paclitaxel. Our results showed that combination therapy led to a more pronounced inhibition of cell viability and increased cell apoptosis. The enhanced efficacy of chemotherapy was attributed to the increased cell permeability induced by cavitation. Importantly, compared with chemotherapy alone (nab-paclitaxel), chemotherapy combined with ultrasound-mediated microbubble cavitation significantly inhibited tumor growth and prolonged the survival of tumor-bearing mice in an orthotopic mouse model of RM-1 prostate carcinoma, indicating the synergistic effects of combined therapy on tumor reduction. Furthermore, we analyzed tumor-infiltrating lymphocytes and found that during chemotherapy, the proportions of CTLA4^+^ cells and PD-1^+^/CTLA4^+^ cells in CD8^+^ T cells slightly increased after cavitation treatment.

## Highlights

• Combination therapy increased cell apoptosis and the inhibition of cell viability.
• Combination therapy enhanced chemotherapy efficacy by increasing cell permeability.
• Success in developing an orthotopic model of prostate tumor implantation in mice.
• Combination therapy inhibited tumor growth and prolonged the survival of mice.

## 1 Introduction

In the current treatment of prostate cancer, the majority of patients with localized prostate cancer received radical prostatectomy and 20–30% of the patients experienced biochemical recurrence (BCR) after radical prostatectomy (RP) during follow-up [1,2]. For the patients who experience biochemical recurrence, endocrine therapy is an important and effective non-surgical treatment for prostate cancer. But with disease progression, patients will inevitably enter the stage of castration resistance. Chemotherapy is an important treatment for castration-resistant prostate cancer (CRPC), especially metastatic CRPC (mCRPC). In recent years, the chemotherapy regimen for prostate cancer has been constantly optimized, and the indications for chemotherapy have been gradually expanding [4,5]. Nevertheless, the treatment outcomes of chemotherapy are not satisfactory, and the systemic toxicity of chemotherapeutic agents limits their use in the treatment of prostate cancer [6,7].

Sufficient drug concentration in tumor cells is a necessary condition for effective chemotherapy. The blood-prostate barrier, which is a unique barrier structure in the prostate, and the inherent biological barrier in the tumor microenvironment may hinder the delivery of chemotherapeutics to prostate cancer cells [8,9]. The blood-prostate barrier may be composed of prostatic ductal epithelial cells, capillary endothelial cells, with a tight junction between them [8,10]. In 2000, for the first time, Fulmer et al. confirmed the existence of the blood-prostate barrier through experiments. They injected radiolabeled ^3^H-dextran (*MW* = 2 × 10^6^), ^14^C-urea (*MW* = 60) and ^3^H-water (*MW* = 18) into adult Sprague-Dawley rats intravenously, and obtained samples of prostatic ductal fluid and arterial blood by micropuncture method. Their study found that 70–80% of ^3^H-water and 50–60% of ^14^C-urea could pass through the prostatic duct epithelium, while ^3^H-dextran of high molecular weight could barely pass through the prostatic duct epithelium (<2%), which confirmed the selective permeability of rat prostatic duct epithelium to substances of different molecular weights [10]. Therefore, Opening the blood-prostate barrier in the prostate gland and the biological barrier in prostate cancer can not only facilitate the delivery of chemotherapeutic drugs to the prostate and tumor, but also reduce the concentration of drugs in the circulation. This is a promising approach for overcoming the challenges associated with prostate cancer chemotherapy.

Ultrasound (US) is not only utilized as a clinical diagnostic tool, but also as a therapeutic method that plays an important role in drug delivery and gene transfection [11,12]. US-induced cavitation can increase the permeability of cells and tissues, and is, therefore, widely used to open biological barriers, such as the blood-brain and blood-pancreas barriers [13,14]. Furthermore, studies have shown that US-induced cavitation can open the blood-prostate barrier, increasing the permeability of prostate tissue, promoting the entry of antibiotics into the prostate, and contributing to the treatment of chronic prostatitis [8,15]. In 2016, a human clinical trial confirmed that US plus microbubbles (MB) enhanced the treatment of inoperable pancreatic cancer with gemcitabine with no additional toxicities [14].

However, few studies have explored the application of cavitation in prostate cancer treatment, and most of the prostate cancer animal models used in these studies were subcutaneous tumor models [16,17]. Due to the presence of the blood-prostate barrier in the prostate, we believe that the subcutaneous tumor model cannot simulate human prostate cancer very well. Studies have shown that the mouse and human prostates have similar tissue structural characteristics at the microscopic level [18]. Theoretically, the orthotopic tumor model is more suitable for studying prostate cancer than the subcutaneous tumor model. In this study, we used RM-1 mouse prostate carcinoma cells to explore the effect of US-induced cavitation on enhancing the efficacy of chemotherapy for the treatment of prostate cancer *in vitro*. And the permeability of cells was detected using calcein. Calcein is a non-permeable fluorescent dye with a molecular weight of 622.5. The molecular weight of PTX is 853.9, similar to that of calcein. Therefore, the entry of calcein into cells was used as an indicator of the cellular uptake of PTX. Furthermore, we developed an orthotopic mouse model of RM-1 prostate cancer in which we innovatively verified the sensitization effect of cavitation on chemotherapy. Prostate cancer is immunologically classified as a “cold” tumor, and has shown poor response to immune-related treatment [19]. Previous studies have reported that cavitation could reverse tumors to an immunoactive microenvironment and is proposed as another potential pathway for immunotherapy [20]. Therefore, we preliminarily explored the effect of cavitation on the immune microenvironment of prostate cancer, providing a theoretical basis for further studies on chemotherapy and immunotherapy combination treatments.

## 2 Materials and methods

### 2.1 Cell line and animals

RM-1 mouse prostate carcinoma cells (androgen-insensitive), which are formed by the oncogene *ras* + *myc* infected with C57BL/6 mice through the replication-deficient retrovirus Zipras/myc9, purchased from the Cell Resource Center, IBMS, CAMS/PUMC (Beijing, China), were cultured in RPMI-1640 medium (Corning, USA) supplemented with 10% fetal bovine serum (FBS) (Hyclone, USA) and 1% penicillin and streptomycin (Gibco, USA) at 37 °C in a humidified 5% CO_2_ atmosphere.

Eight-week-old male C57BL/6 mice, purchased from the Laboratory Animal Department, Peking University Health Science Center, were raised in barrier conditions. All experimental procedures were approved by the Experimental Animal Welfare Ethical Branch of the Biomedical Ethics Committee, Peking University (LA2019316).

### 2.2 US apparatus and MB

US treatment was performed using a VINNO 70 US instrument and an X4-12L ultrasonic probe (Vinno Corporation, Suzhou, China). US irradiation was performed in the VFlash mode of the ultrasound instrument under the following conditions: mechanical index, 0.6; frequency, 3 MHz; pulse repetition frequency, 2000 Hz; pulse length, 26.0 cycles (see Supplementary Material for the selection of ultrasonic parameters).

The SonoVue agent (Bracco, Italy) was used as MB. Prior to use, the SonoVue agent was mixed with 5 mL of normal saline to prepare an MB suspension.

### 2.3 Drugs

Paclitaxel (PTX) (Coolaber, China) was used as the chemotherapeutic agent *in vitro*, whereas nab-paclitaxel (nab-PTX) (CSPC, China) (paclitaxel: albumin, 100 mg: 900 mg) was used *in vivo*. Calcein (Coolaber, China), a non-permeable fluorescent dye, was used as a permeability tracer to evaluate cell permeability.

### 2.4 *In vitro* combination treatment of chemotherapy and cavitation

#### 2.4.1 Treatment

The RM-1 cell suspensions were divided into six treatment groups: the control (untreated), MB (50 μL MB/1 mL of medium), PTX (10 ng PTX/1 mL of medium), PTX+MB (combination of PTX and MB), PTX+US (combination of PTX and US for 30 s, and PTX+US+MB (combination of PTX, US, and MB) groups.

#### 2.4.2 Cell viability

RM-1 cells were seeded in a 96-well plate (4000 cells/well) and accordingly treated. The experiment was performed in triplicates. After treatment, cells were cultured for 72 h, and cell viability was determined using the CCK-8 assay. After medium replacement with 100 μL fresh medium/well, a total of 10 μL CCK-8 reagent (Dojindo, Japan) was added into each well according to the manufacturer's instructions, including three blank wells (100 μL medium and 10 μL CCK-8 reagent without cells), followed by incubation for another 2 h. The absorbance in each well was measured at 450 nm using a microplate reader (Thermo, USA), and cell viability was calculated based on the absorbance.

#### 2.4.3 Cell apoptosis

RM-1 cells were seeded in a 6-well plate (1 × 10^6^ cells/well) and accordingly treated. After 72 h, the Annexin V/7-AAD apoptosis detection kit (Biolegend, USA) was used to detect cell apoptosis. Annexin V and 7-AAD double staining was carried out according to the manufacturer's instructions. Cell apoptosis was detected using a CytoFLEX S flow cytometer (Beckman Coulter, USA).

### 2.5 Effect of cavitation on RM-1 cells

RM-1 cells were cultured in 24-well plates (5 × 10^4^ cells/well) and divided into four treatment groups: the control, MB, US, and US+MB groups. For cell permeability analysis, calcein (1 μL in 1 mL of medium) was added to each sample prior to US treatment. After 30 s of US exposure, the cells were placed in the dark for 2 min and then washed 3–4 times with PBS. Fluorescence microscopy (Nikon, Japan) and CytoFLEX S flow cytometry were used to investigate the cellular uptake of calcein. For analysis of cell viability and apoptosis, the cells were cultured for 72 h after treatment. Cell viability and apoptosis were then assessed.

### 2.6 *In vivo* mouse experiments

#### 2.6.1 Mice and orthotopic tumor models

To prepare cells for intraprostatic injection, RM-1 cells in the logarithmic growth phase were harvested and suspended in an 1:3 mixture of Matrigel (BD, USA) and RPMI-1640 medium at a density of 1 × 10^7^ cells/mL. Mice were anesthetized using an intraperitoneal injection of tribromoethanol (Sigma, USA) (200 mg/kg). Then, the mice were then fixed in the supine position on the operating table, and the skin and abdominal muscles were opened using a 10 mm midline incision in the lower abdomen. The prostate could be seen below the bladder by gently lifting the bladder to the head. A 10 μL suspension was slowly injected into the prostate using a 10 μL microsyringe. After removing the needle, a cotton swab was placed on the injection site for 30 s to avoid suspension leakage. The bladder was placed back into the abdominal cavity, and the incision was closed with sutures. The mice were returned to their cages and observed until awake.

#### 2.6.2 Treatment and survival analysis

Tumor-bearing mice were divided into four groups: the control, US+MB, PTX, and PTX+US+MB groups. For the control group, normal saline was injected into each mouse via the tail vein. For the US+MB group, an 1:1 (v/v) mixture of MB and normal saline was injected. In the PTX group, nab-PTX was administered. For the PTX+US+MB group, an 1:1 (v/v) mixture of MB and nab-PTX was injected. The PTX dose was 12 μg/g/mouse in the PTX and PTX+US+MB groups. Each mouse was exposed to US or sham US (US equipment off) for 3 min. Treatment was administered three times on the 8^th^, 11^th^ and 14^th^ days after tumor bearing. Before treatment, the mice were anesthetized using an intraperitoneal injection of tribromoethanol, and the hair on their lower abdomen was removed.

Survival was calculated as the time from the first treatment to death. Mice that died before the third treatment were excluded from the survival analysis, and such deaths were not considered to be treatment-related.

#### 2.6.3 Analysis of tumor growth and tumor-infiltrating lymphocytes

Animals were sacrificed on the 3^rd^ day after the last treatment, and the tumors were surgically excised. The tumor size was measured using a caliper, and the tumor volume was calculated as volume = length × width^2^/2.

After removing the necrotic tissue, the tumor tissue was cut into pieces in the medium. The tumor fragments were digested using a gentleMACS^TM^ tissue dissociator (Miltenyi Biotec, Germany) and tissue dissociation kits (Miltenyi Biotec, Germany). Lymphocytes were extracted using a mouse lymphocyte separation liquid (Dakewe, China) after passing the homogenate through a cell strainer of 70 μm pores (Corning, USA). The cells were then labeled with antibodies. Anti-CD3, anti-CD45, anti-CD4, anti-CD8, anti-PD-1, anti-CTLA4 and anti-Foxp3 antibodies were used (BioLegend, USA). The cells were then detected using CytoFLEX S flow cytometry. All experimental procedures were performed according to the manufacturer's instructions.

### 2.7 Statistical analyses

Statistical analyses were performed using GraphPad Prism (version 7.0) and SPSS (version 20.0). One-way analysis of variance was used to compare different groups. Kaplan-Meier curves and log-rank tests were used for survival analysis. A *P* value of <0.05 was considered statistically significant.

## 3 Results

### 3.1 US-mediated MB cavitation enhanced the efficacy of chemotherapy *in vitro*

**Fig. 1:**
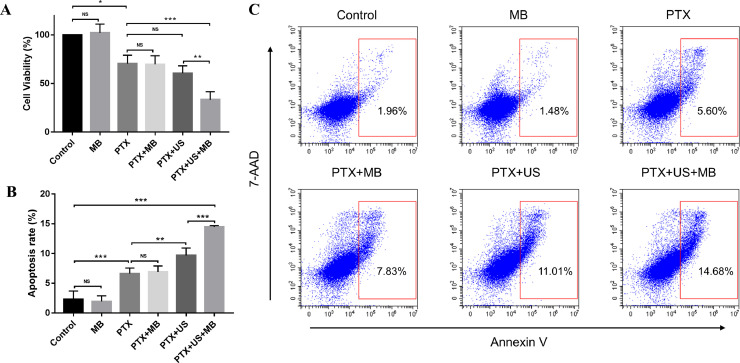
US-mediated MB cavitation enhanced the efficacy of chemotherapy *in vitro.* (A) Histogram of cell viability after different treatments. (B and C) Comparison of cell apoptosis after different treatments. US, ultrasound; MB, microbubbles; PTX, paclitaxel; * *P* < 0.05; ** *P* < 0.01; *** *P* < 0.001; NS: non-significant.

Compared with the control group, PTX treatment significantly induced tumor cell death (Fig. 1A). Among all the groups, the cell viability of the PTX+US+MB group was the lowest.

To assess the apoptosis rate, we stained cells for 7-AAD and Annexin V. As expected, PTX treatment increased tumor cell apoptosis (Fig. 1B and C). However, there was no significant difference in the apoptosis rate between the PTX+MB and PTX groups (*P* = 0.750), while the apoptosis rate of the PTX+US group was slightly higher than that of the PTX group (*P* = 0.003). Interestingly, the PTX+US+MB group had the highest cell apoptosis rate, suggesting that US-mediated MB cavitation significantly increased the chemotherapy-induced cell apoptosis.

### 3.2 US-mediated MB cavitation increased cell permeability, without affecting cell viability and apoptosis

**Fig. 2:**
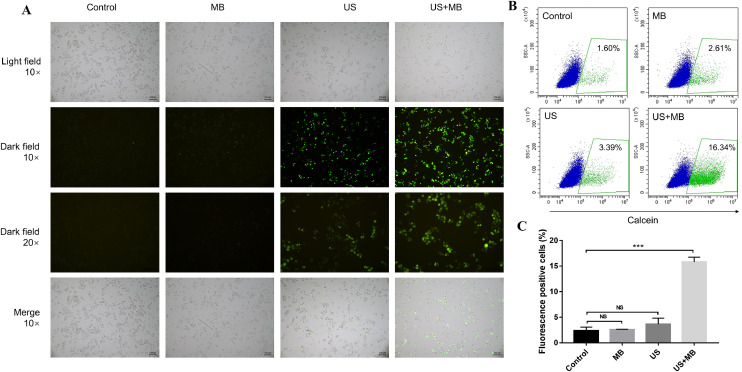
US-mediated MB cavitation increased cell permeability. (A) Fluorescence images of calcein-postive cells. (B and C) Flow cytometry results of calcein-positive cells. US, ultrasound; MB, microbubbles; *** *P* < 0.001; NS: non-significant.

To explore whether US+MB could enhance cell permeability, calcein was added to the cell culture. Neither MB nor US could increase RM-1 cell permeability, while treatment of RM-1 cells with US plus MB markedly enhanced cell permeability (Fig. 2A). Furthermore, we measured the calcein-positive cells using flow cytometry. Treatment with US+MB resulted in the highest number of calcein-positive cells, further confirming that US-mediated MB cavitation could increase cell permeability (Fig. 2B and 2C).

**Fig. 3:**
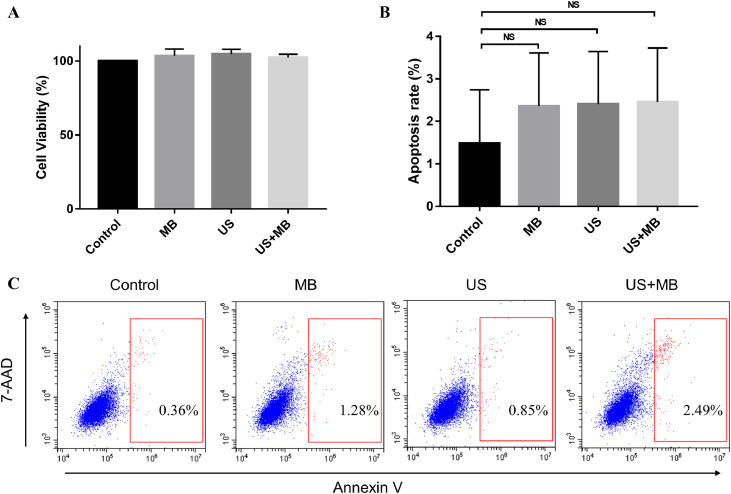
US-mediated MB cavitation did not affect cell viability and apoptosis. (A) Histogram of cell viability after different treatments. (B and C) Comparison of cell apoptosis after different treatments. US, ultrasound; MB, microbubbles; NS: non-significant.

However, we did not observe a significant difference in cell apoptosis among the four groups (Fig. 3B and 3C). Similar results were found regarding cell viability (Fig. 3A). Taken together, treatment with US+MB increased cell permeability, without affecting cell apoptosis and death.

### 3.3 US-mediated MB cavitation enhanced the efficacy of chemotherapy *in vivo*

**Fig. 4:**
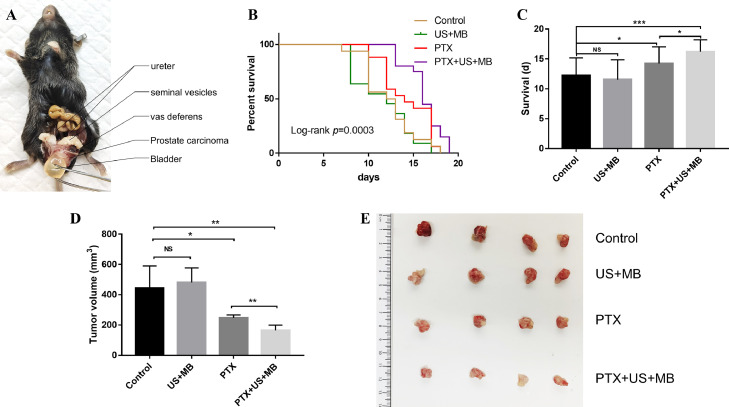
US-mediated MB cavitation enhanced the efficacy of chemotherapy *in vivo.* (A) The orthotopic mouse model of RM-1 prostate carcinoma. (B) Kaplan-Meier curves of survival for tumor-bearing mice in different groups. (C) The comparison of the mean survival day of mice in each treatment group. (D and E) The comparison of tumor volume in each treatment group. US, ultrasound; MB, microbubbles; PTX, nab- paclitaxel; * *P* < 0.05; ** *P* < 0.01; *** *P* < 0.001; NS: non-significant.

To investigate whether treatment with PTX+US+MB could control tumor growth and enhance the survival rate, we successfully established an orthotopic mouse model of RM-1 prostate carcinoma. The anatomical structure of the tumor model was clear. The tumor was located below the bladder with the attachment of both seminal vesicles and vas deferens on the dorsal side (Fig. 4A). Through abdominal dissection, we found that initial tumor growth could be observed at day 8 or day 9 after tumor inoculation (Supplementary Fig. S1).

The Kaplan-Meier curves showed that three cycles of combination therapy prolonged the survival of RM-1 prostate cancer-bearing mice (Fig. 4B). The comparison of the mean survival day of mice in each treatment group showed that there was no significant difference between the US+MB and control groups (*P* = 0.507). However, the mean survival time of the PTX+US+MB group was significantly longer than that of the control group (*P* < 0.001). Importantly, treatment with PTX+US+MB prolonged the survival of the tumor-bearing mice compared with that in the PTX group (*P* = 0.031) (Fig. 4C), indicating that US-mediated MB cavitation further enhanced the efficacy of PTX therapy.

In addition, we monitored the tumor size. As shown in Fig. 4D and 4E, the tumor volume in the US+MB group was similar to that in the control group (*P* = 0.687). As expected, PTX treatment led to slower tumor growth compared to that of the control group (*P* = 0.037). Notably, compared to PTX alone, the combination of US+MB further inhibited tumor growth (*P* = 0.005), suggesting that the combined therapy did elicit a synergistic effect on tumor reduction compared with PTX or US+MB alone.

### 3.4 US-mediated MB cavitation affected the tumor immune microenvironment

After tumor-infiltrating lymphocytes were extracted, we analyzed PD-1 and CTLA4 expression on CD8^+^ T cells and Foxp3 expression in CD4^+^ T cells and CTLA4 expression on CD4^+^ T cells in the control, PTX, and PTX+US+MB groups. The proportion of CTLA4^+^ and PD-1^+^/CTLA4^+^ cells among CD8^+^ T cells was slightly higher in the PTX+US+MB group compared to that in the PTX group, although the difference was not statistically significant (Supplementary Fig. S2A). There was no significant difference in the expression of Foxp3 in CD4^+^ T cells and CTLA4 on CD4^+^ T cells between the PTX and PTX+US+MB groups (Supplementary Fig. S2B).

## 4 Discussion

Chemotherapy is an important treatment for advanced prostate cancer, but previous studies have shown that its efficacy is relatively limited [6,7]. Since its discovery, ultrasonic cavitation has been widely studied in the fields of drug delivery, gene transfection, and tumor treatment [12,21,22]. We investigated the effect of US-induced cavitation on enhancing the efficacy of chemotherapy for the treatment of prostate cancer and preliminarily explored its effect on the tumor immune microenvironment. To our knowledge, this is the first study using an orthotopic animal model of prostate cancer.

Docetaxel is a first-line chemotherapeutic agent commonly used in CRPC, however, its efficacy is relatively limited [3]. Albumin-bound paclitaxel, also known as nab-paclitaxel, is a solvent-free formulation of paclitaxel, and can overcome toxicities associated with the solvents used in the formulation of standard paclitaxel [23]. Due to the albumin component of nab-paclitaxel, it is assumed that the drug could cross endothelial cell monolayers and enter tumors using endogenous albumin transport pathways [24,25]. Further, nab-paclitaxel has also been used in previous studies of ultrasound adjuvant chemotherapy for prostate cancer [17]. Therefore, paclitaxel was used as the chemotherapeutic agent *in vitro*, whereas nab-paclitaxel was chosen *in vivo* in the current study. The prostate cancer cell line used in this study was RM-1 mouse prostate carcinoma cells, which are androgen-insensitive. RM-1 cells are formed by the oncogene *ras* + *myc* infected with C57BL/6 mice through the replication-deficient retrovirus Zipras/myc9 [26]. Therefore, there is no immune rejection between RM-1 cells and C57BL/6 mice, and orthotopic mouse model of RM-1 prostate cancer is suitable for tumor chemotherapy and immune-related studies.

MB are tiny bubbles that are usually composed of inert gas wrapped in albumin, lipid macromolecules, surfactants, and degradable polymer materials [27]. Adding MB to a liquid is equivalent to artificially increasing the number of cavitation nuclei in the liquid. With an increase in the number of tiny bubbles, the threshold of energy required to induce cavitation is reduced, and the intensity of cavitation is obviously enhanced [28]. In our study, MB treatment did not affect cell viability or apoptosis, which proved the safety of MB. US treatment only slightly enhanced the chemotherapeutic effect of PTX on RM-1 cells. However, the chemotherapeutic effect of PTX on RM-1 cells was significantly enhanced when combined with US+MB. Sorace et al. found that compared with chemotherapy alone, chemotherapy combined with US+MB increased 2LMP breast cancer cell mortality by 50%. Further studies found that the permeability of 2LMP cells significantly increased after combined treatment with US+MB [29]. Wang et al. irradiated a suspension of DU145 human prostate cancer cells containing MB and mitoxantrone using US and found that this enhanced the mitoxantrone-induced inhibition of cell proliferation [30]. These results are consistent with those of our experiments that showed that US-mediated MB cavitation enhanced the effect of chemotherapy *in vitro*.

To verify the mechanism underlying the chemotherapy-enhancing effects of cavitation on RM-1 cells, we assessed cell permeability, cell viability, and apoptosis following treatment with US+MB. We found that US-mediated MB cavitation significantly increased the permeability of RM-1 cells. This has also been verified in other studies [31,32]. Furthermore, we found that US treatment with or without MB did not affect cell viability or apoptosis. However, an *in vitro* study conducted by Maeshige et al. reported that PC-3 and LNCaP cells proliferation was significantly inhibited by US irradiation (3.0 W/cm^2^, 3 MHz) [33]. This seems to be inconsistent with our results. Ward et al. found that lethal sonoporation and reparable sonoporation were observed in cells treated with cavitation of different intensities. The reparable sonoporation was reversible, and cell permeability increased transiently. Lethal sonoporation can result in lethal cell damage [34]. In our study, the acoustic power of the VINNO 70 US instrument was less than 720 mW/cm^2^. Compared to that in Maeshige's study, the cavitation intensity in our study was relatively lower, which could be the reason cell viability and apoptosis were not affected by US. Thermal effect is another important effect induced by ultrasound, and previous literature has reported that elevated temperature can induce tumor death or apoptosis [35]. Thermal effect is related to the intensity of ultrasound and the Thermischer Index (TI) [36]. In this study, the acoustic power of the US instrument was less than 720 mW/cm^2^ and TI was approximately 0.1. Thus, the increase in temperature is negligible. Therefore, we speculated that US-mediated MB cavitation increased the effect of chemotherapy in prostate cancer cells by increasing RM-1 cell permeability and improving the cellular uptake of PTX.

To further prove that US+MB could enhance chemotherapy in prostate cancer, we conducted animal experiments. Most previous relevant studies were performed on subcutaneous tumor-bearing mouse models [17,37] which lacked the blood-prostate barrier. The classical seed and soil hypothesis suggests that tumor growth and metastasis depend on suitable soil (microenvironment) [38]. Furthermore, the mouse prostate has been shown to be similar to the human prostate at the microscopic level [18]. Therefore, we used an orthotopic prostate cancer model, which is speculated to have a blood-prostate barrier. The survival of mice with prostate cancer was prolonged, and tumor growth was inhibited after three cycles of combination therapy. Wamel et al. developed acoustic cluster therapy (ACT), a novel approach for ultrasound-mediated drug delivery [39]. They investigated ACT in combination with nab-PTX for the treatment of subcutaneous prostate cancer in nude mice [17] and found that the survival of mice in the ACT+nab-PTX group was significantly longer than that in the other groups, and that tumor growth was also significantly inhibited in this group. The conclusions of their research are in line with ours. Wang et al. found that low frequency US +MB inhibited the growth of prostate tumors in nude mice and attributed the effect to cavitation-induced microvessel destruction [40]. However, the oncological outcomes were similar between the US+MB and control groups in our study. Therefore, following a comprehensive analysis of the results of cell and animal experiments, we deduced that US-mediated MB cavitation could increase prostate and tumor permeability, and that this could be the mechanism by which cavitation induced an increase in chemotherapeutic efficacy in the present study.

The tumor microenvironment of solid tumors not only includes tumor cells, but also normal cells, such as stromal and immune cells, which play an important role in tumor growth and progression [41,42]. Unfortunately, the results of clinical studies have shown that the efficacy of immune checkpoint inhibitors in the treatment of prostate cancer is not satisfactory [43,44]. At present, few studies have explored cavitation and tumor immunity in prostate cancer. The tumor models used in previous studies were mostly nude mouse prostate cancer models, which limited the exploration of tumor immunity [17,37]. Thus, our tumor model, based on C57B/L6 mice, which have an intact immune system, was suitable for exploring the effect of cavitation on the immune microenvironment of prostate cancer. PD-1 and CTLA4 are widely studied immune checkpoints. PD-1 is expressed on the surface of T cells, B cells, and NK cells. The combination of PD-1 and PD-L1, a ligand expressed on the tumor surface, can attenuate the activity of PD-1^+^ cells, inhibit their proliferation, and induce their apoptosis, thereby mediating tumor immune escape. Moreover, blocking the PD-1/PD-L1 pathway can enhance the anti-tumor immune response [45]. Similarly, the interactions of ligands with CTLA4 inhibit T-cell responses [46]. In our study, in the presence of chemotherapy, the proportions of CTLA4^+^ and PD-1^+^/CTLA4^+^ cells among CD8^+^ T cells slightly increased after combined treatment with US+MB. Although the differences were not statistically significant, our results suggest that it is feasible to combine immune checkpoint inhibitors, chemotherapeutic agents, and cavitation in the treatment of prostate cancer. Furthermore, cavitation can increase the permeability of prostate cancer, thereby facilitating the targeted delivery of immune checkpoint inhibitors to tumors. Therefore, triple-combination therapy has the potential to improve the treatment efficacy for prostate cancer. However, the efficacy of this combination therapy needs to be verified in further experiments, which is the direction of our future research.

Our study has several limitations. First, in our *in vivo* experiments, we focused on analyzing mouse survival and tumor growth and did not study tumor metastasis. Second, the mechanism by which cavitation enhances the chemotherapeutic effect and regulates the immune microenvironment requires further study. Third, as this study is a preclinical study, prospective clinical trials are urgently needed. In conclusion, we confirmed that US-mediated MB cavitation can enhance the efficacy of chemotherapy for the treatment of prostate cancer by increasing the membrane permeability of the prostate gland, and verified the efficacy of this treatment in an orthotopic tumor model of prostate cancer. Furthermore, we found that US-mediated MB cavitation has a tendency to upregulate the expression of PD-1 and CTLA4 on the surface of CD8^+^ T cells in the presence of chemotherapy.

## Authors’ contributions

Haizhui Xia and Decao Yang conceived and designed the study, performed experiments, analyzed data, drafted and revised the manuscript.

Wei He, Xuehua Zhu and Zenan Liu performed some animal experiments.

Ye Yan designed the study and revised the manuscript.

Tong Liu, Jianling Yang, Shi Tan, Jie Jiang and Xiaofei Hou conceived and designed the study.

Huile Gao analyzed data, reviewed and revised the manuscript.

Ling Ni performed experiments, analyzed data, reviewed and revised the manuscript.

Jian Lu conceived and designed the study, reviewed and revised the manuscript.

## Appendix Supplementary materials

Image, application 1

Image, application 2
