# Supplementary material for: Ultrasound-mediated microbubbles cavitation enhanced chemotherapy of advanced prostate cancer by increasing the permeability of blood-prostate barrier
Source: Transl Oncol. 2021 Jul 13;14(10):101177. doi: 10.1016/j.tranon.2021.101177 (PMC8287239; doi:10.1016/j.tranon.2021.101177)
Supplement: Supplementary file 1 [file mmc1.docx]

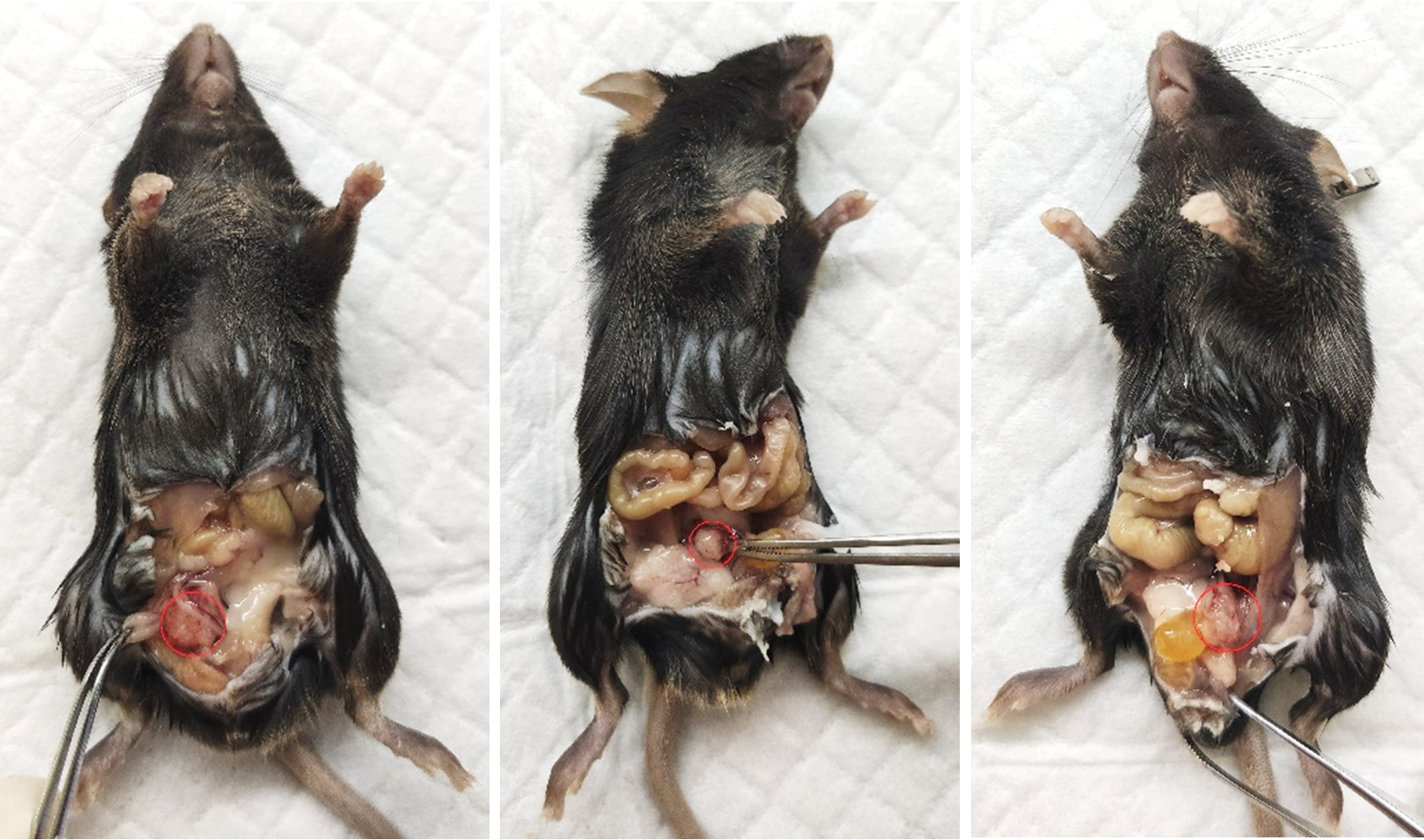


**Supplementary Fig. S1 -** The local tumor situation in the mouse prostate on the 9^th^ day after tumor-bearing.


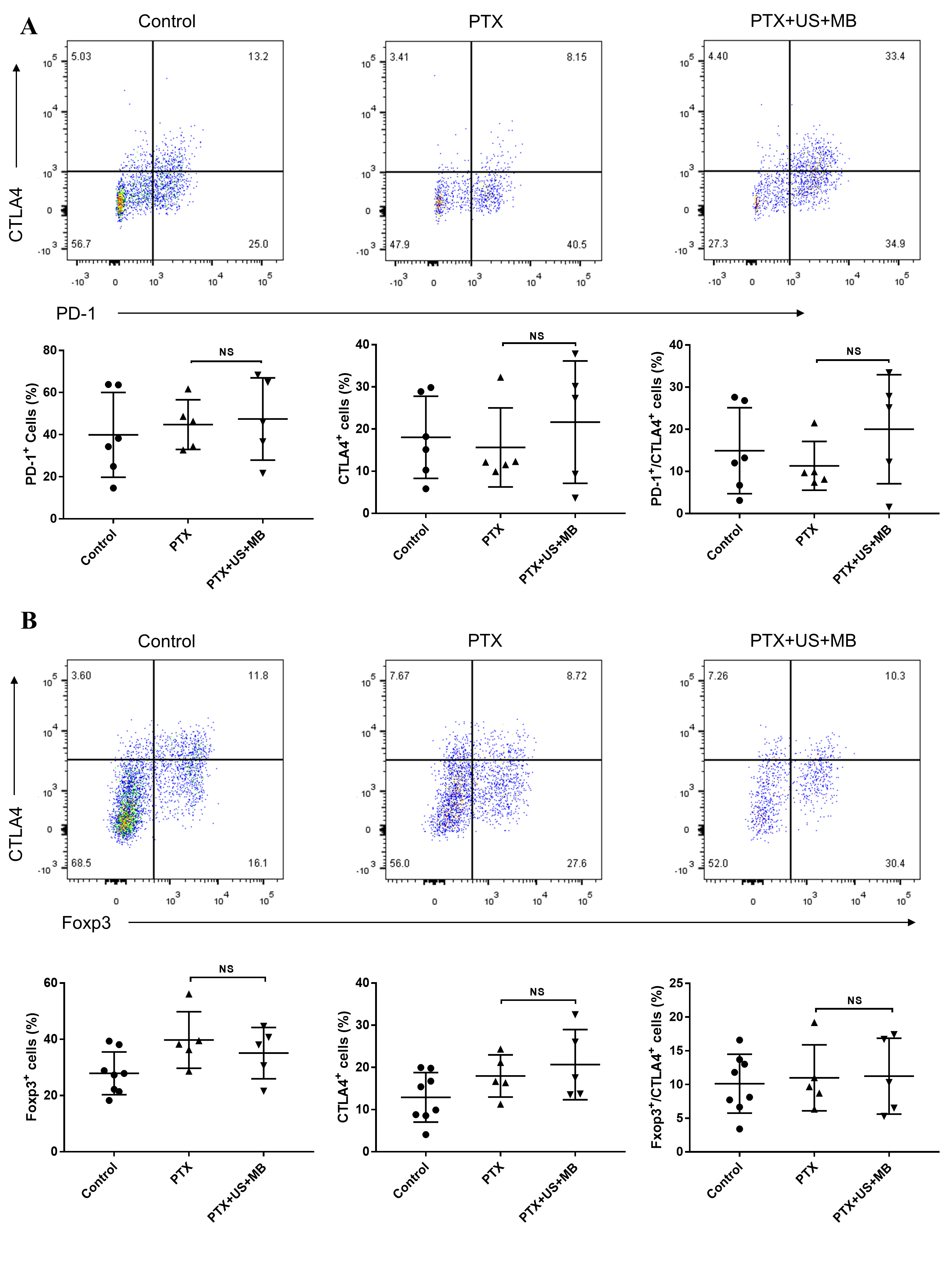


**Supplementary Fig. S2 -** US-mediated MB cavitation affected the tumor immune microenvironment. (A) Expression of PD-1 and CTLA4 on CD8^+^ T cells in tumor after chemotherapy with or without US+MB. (B) Expression of Foxp3 in CD4^+^ T cells and CTLA4 on CD4^+^ T cells in tumor after chemotherapy with or without US+MB. US, ultrasound; MB, microbubbles; PTX, nab- paclitaxel; NS: non-significant.
