# Supplementary material for: Ultrasound-mediated microbubbles cavitation enhanced chemotherapy of advanced prostate cancer by increasing the permeability of blood-prostate barrier
Source: Transl Oncol. 2021 Jul 13;14(10):101177. doi: 10.1016/j.tranon.2021.101177 (PMC8287239; doi:10.1016/j.tranon.2021.101177)

While maintaining the consistency of other ultrasound parameters, RM-1 cell suspensions containing microbubbles and paclitaxel were irradiated with ultrasound at different frequencies (3 MHz, 4 MHz and 5 MHz) (Fig. A) or mechanical index (0.2, 0.4 and 0.6) (Fig. B). After treatment, the cells were cultured for 72 h, and then cell viability was determined by CCK-8 assay. The cell viability in 3 MHz group was significantly lower than that in 4 MHz group (*P*<0.001) and 5 MHz group (*P*<0.001) and it was significantly lower when MI was 0.6 than MI was 0.2 (*P*<0.001) and 0.4 (*P*=0.001). Therefore, the ultrasonic frequency used in the experiment was determined to be 3 MHz and the mechanical index was 0.6.


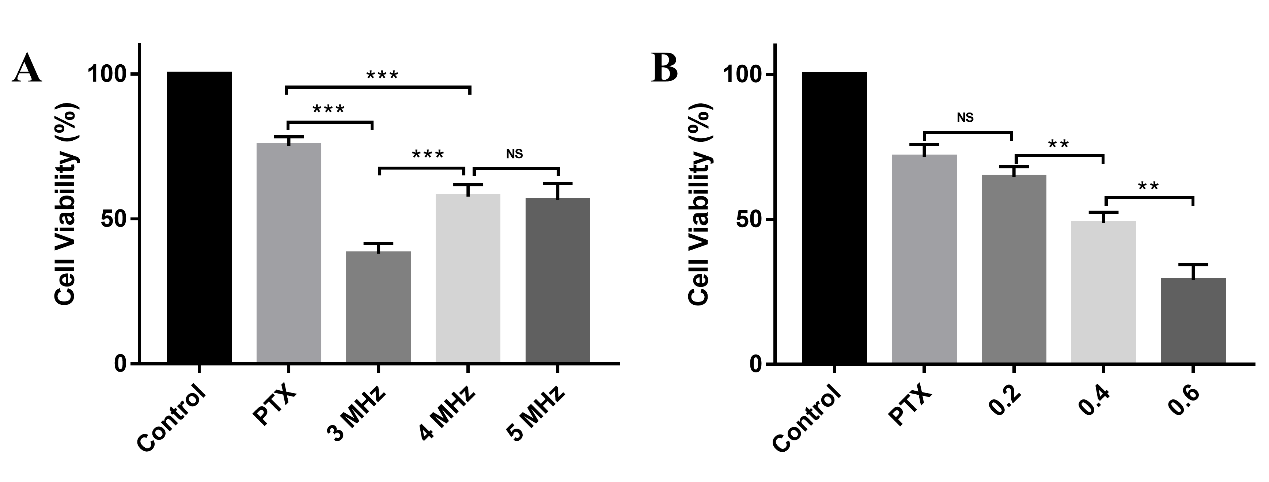

Supplement: Supplementary file 2 [file mmc2.docx]
